# Assessment of Regional Nursing Home Preparedness for and Regulatory Responsiveness to Wildfire Risk in the Western US

**DOI:** 10.1001/jamanetworkopen.2023.20207

**Published:** 2023-06-26

**Authors:** Natalia Festa, Kaitlin Fender Throgmorton, Kendra Davis-Plourde, David M. Dosa, Kai Chen, Emma Zang, Jill Kelly, Thomas M. Gill

**Affiliations:** 1 Department of Internal Medicine, Yale School of Medicine, New Haven, Connecticut; 2 National Clinician Scholars Program at Yale University, New Haven, Connecticut; 3 Harvey Cushing/John Hay Whitney Medical Library, School of Medicine, Yale University, New Haven, Connecticut; 4 Department of Biostatistics, Yale School of Public Health, New Haven, Connecticut; 5 School of Public Health, Brown University, Providence, Rhode Island; 6 Warren Alpert Medical School, Brown University, Providence, Rhode Island; 7 Center of Innovation for Long-Term Services and Supports, Providence Veterans Affairs Medical Center, Providence, Rhode Island; 8 Department of Environmental Health Sciences, Yale School of Public Health, New Haven, Connecticut; 9 Department of Sociology, Yale University, New Haven, Connecticut

## Abstract

**Importance:**

It is uncertain whether emergency preparedness and regulatory oversight for US nursing homes are aligned with local wildfire risk.

**Objective:**

To evaluate the likelihood that nursing homes at elevated risk of wildfire exposure meet US Centers for Medicare & Medicaid Services (CMS) emergency preparedness standards and to compare the time to reinspection by exposure status.

**Design, Setting, and Participants:**

This cross-sectional study of nursing homes in the continental western US from January 1, 2017, through December 31, 2019, was conducted using cross-sectional and survival analyses. The prevalence of high-risk facilities within 5 km of areas at or exceeding the 85th percentile of nationalized wildfire risk across areas overseen by 4 CMS regional offices (New Mexico, Mountain West, Pacific/Southwest, and Pacific Northwest) was determined. Critical emergency preparedness deficiencies cited during CMS Life Safety Code Inspections were identified. Data analysis was performed from October 10 to December 12, 2022.

**Main Outcomes and Measures:**

The primary outcome classified whether facilities were cited for at least 1 critical emergency preparedness deficiency during the observation window. Regionally stratified generalized estimating equations were used to evaluate associations between risk status and the presence and number of deficiencies, adjusted for nursing home characteristics. For the subset of facilities with deficiencies, differences in restricted mean survival time to reinspection were evaluated.

**Results:**

Of the 2218 nursing homes in this study, 1219 (55.0%) were exposed to elevated wildfire risk. The Pacific/Southwest had the highest percentage of both exposed (680 of 870 [78.2%]) and unexposed (359 of 486 [73.9%]) facilities with 1 or more deficiencies. The Mountain West had the largest difference in the percentage of exposed (87 of 215 [40.5%]) vs unexposed (47 of 193 [24.4%]) facilities with 1 or more deficiencies. Exposed facilities in the Pacific Northwest had the greatest mean (SD) number of deficiencies (4.3 [5.4]). Exposure was associated with the presence of deficiencies in the Mountain West (odds ratio [OR], 2.12 [95% CI, 1.50-3.01]) and the presence (OR, 1.84 [95% CI, 1.55-2.18]) and number (rate ratio, 1.39 [95% CI, 1.06-1.83]) of deficiencies in the Pacific Northwest. Exposed Mountain West facilities with deficiencies were reinspected later, on average, than unexposed facilities (adjusted restricted mean survival time difference, 91.2 days [95% CI, 30.6-151.8 days]).

**Conclusions and Relevance:**

In this cross-sectional study, regional heterogeneity in nursing home emergency preparedness for and regulatory responsiveness to local wildfire risk was observed. These findings suggest that there may be opportunities to improve the responsiveness of nursing homes to and regulatory oversight of surrounding wildfire risk.

## Key Points

**Question:**

Are nursing homes at elevated risk of wildfire exposure likelier to meet federal emergency preparedness standards or subject to greater regulatory oversight?

**Findings:**

In this cross-sectional study of 2218 nursing homes in the western US, regional heterogeneity in nursing home emergency preparedness for wildfire episodes was observed, and facilities at elevated exposure risk in the Mountain West and Pacific Northwest had poorer compliance with emergency preparedness criteria than unexposed facilities. Of the noncompliant facilities, exposed nursing homes in the Mountain West incurred longer times to reinspection than their unexposed counterparts.

**Meaning:**

These findings suggest that there are opportunities to improve the regional responsiveness of nursing homes and regulatory oversight to surrounding environmental hazards.

## Teaser

This cross-sectional study assesses emergency preparedness for and regulatory responsiveness to wildfires among nursing homes at elevated wildfire risk in the western US.

## Introduction

Because nursing homes are affected by a confluence of factors that increase resident vulnerability, their adaptation to environmental hazards, such as wildfires, is a federal priority.^1^ The US Centers for Medicare & Medicaid Services (CMS) published its Emergency Preparedness Requirements for Medicare & Medicaid Participating Providers and Suppliers in 2016, setting the standards to which nursing homes, among other providers, are subject during emergency preparedness inspections.^2^ This rule encourages nursing homes to adopt an all-hazards approach to emergency preparedness, under which facilities are responsible for appraising and preparing for potential risks, including environmental hazards. While the intentional flexibility of the all-hazards framework promotes context-responsive emergency planning without imposing an undue regulatory burden,^3,4^ recent federal audits have demonstrated patterns of emergency preparedness deficiencies that are misaligned with prominent environmental risks.^5,6^ Because the probability of weather conditions that are conducive to wildfire episodes has been increasing in the US,^7,8^ nursing home preparedness for wildfires is of particular importance in regions where such episodes are common.

While federal audits offer vital details regarding nursing home emergency preparedness, these reports are often constrained to a small subset of facilities within individual states.^5^ For this reason, relatively little is known about the regional alignment between specific environmental hazards and nursing home emergency preparedness or patterns of regulatory oversight.^9^ Improved understanding of such regional relationships is important because CMS regional offices (ROs) oversee state enforcement actions and standardized compliance with federal emergency preparedness criteria.^4^ Ensuring that nursing homes are prepared to respond to surrounding environmental hazards will become increasingly important if the frequency of community exposure to wildfires continues to increase, as has been projected.^10^ Direct exposure to heat and particulate matter from wildfire episodes places residents at heightened risk of morbidity and mortality.^11,12^ Moreover, inadequate nursing home preparedness for wildfires may contribute to resident abandonment and evacuation to non–health care settings.^13,14,15^

In this study, we assessed whether emergency preparedness by US nursing homes, as measured by adherence to CMS standards, is commensurate with facilities’ risk of wildfire exposure. Further, we evaluated whether patterns of regulatory oversight differ according to facilities’ wildfire exposure risk across CMS regulatory regions. We hypothesized that nursing homes with the highest exposure risk would exhibit greater compliance with CMS emergency preparedness standards compared with lower-risk facilities. We also hypothesized that high-risk nursing homes with documented emergency preparedness deficiencies would undergo CMS Life Safety Code (LSC) reinspection sooner than lower-risk facilities. To accomplish our objectives, we estimated regionally stratified associations between location in an area of heightened wildfire risk and compliance with CMS emergency preparedness standards. We also evaluated regionally stratified associations between wildfire exposure and time to LSC reinspection for facilities with critical emergency preparedness deficiencies. The results of this study should inform policies to better align nursing home emergency preparedness and regulatory oversight with local wildfire risk within and across regulatory regions.

## Methods

This cross-sectional study did not involve human participants and was deemed exempt from review and informed consent by the Yale University Institutional Review Board under 45 CFR §46.104. The study followed the Strengthening the Reporting of Observational Studies in Epidemiology (STROBE) reporting guideline.

### Data Sources and Sampling Approach

We used the CMS Provider Information catalog to identify certified nursing homes within the continental western US, where wildfires are most common; 2357 such nursing homes were found.^16^ These homes included facilities in Arizona, California, Colorado, Idaho, Montana, Nevada, New Mexico, Oregon, Utah, Washington, and Wyoming. We evaluated facilities’ emergency preparedness deficiencies based on inspections from January 1, 2017, to December 31, 2019. We omitted 139 nursing homes because they had missing facility characteristics or location data during the observation window, leading to a final analytic sample of 2218 facilities.

### Exposure Definition

We used the third edition of the US Wildfire Hazard Potential Index (WHPI) to evaluate wildfire potential using local landscape conditions.^17^ The WHPI contains information regarding local wildfire hazard potential at a spatial resolution of 270 m^2^, based on simulations under tens of thousands of hypothetical landscape and meteorological conditions to estimate burn probability within each 270-m^2^ pixel.^17,18,19^ The models consider landscape conditions circa 2014 and were updated in 2020.^18,19^

We defined high-risk (exposed) nursing homes as those located within 5 km of areas at or exceeding the 85th percentile of nationalized wildfire hazard potential for the US. This working definition of exposure status reflects risk thresholds and does not necessarily indicate direct exposure to a wildfire episode during the observation period. The primary risk threshold corresponds to cut points for high (85th to 95th percentiles) and very high (96th to 99th percentiles) nationalized wildfire hazard potential within the WHPI.^18^ We refrained from defining separate percentile thresholds for each state because such an approach would understate wildfire risk to facilities in states with higher average risk, which are concentrated in the western US.

### Outcome Measures

We compiled emergency preparedness deficiencies using CMS LSC inspections over 3 years from January 2017 to December 2019.^20^ We used emergency preparedness deficiencies as a proxy for nursing home emergency preparedness. From the 252 potential emergency preparedness deficiencies, we selected a subset deemed to be most critical in accordance with prior literature and emergency preparedness guidance (eTable 1 in Supplement 1).^9,21,22,23^ We also compiled information regarding the scope and severity of each deficiency, using the following categories defined by CMS (from least to most severe): (1) no actual harm with potential for minimal harm, (2) no actual harm with potential for more than minimal harm, (3) actual harm that is not immediate jeopardy, and (4) immediate jeopardy to resident health or safety.^24^

We defined 3 outcome measures. Our primary outcome classified whether facilities were cited for at least 1 critical emergency preparedness deficiency during the observation window. Our second outcome summed the total number of critical emergency preparedness deficiencies per inspection. Our third outcome quantified the mean time to reinspection for nursing homes with documented critical emergency preparedness deficiencies cited during their first inspection within the observation window.

### Facility Characteristics

We used the CMS Provider Information files to evaluate the size (number of beds), ownership status (proprietary vs nonprofit or government ownership), and CMS 5-star ratings for the Staffing, Health Inspection, and Quality domains. The CMS ratings range from 1 to 5, with higher scores indicating better performance. We used LTCFocus (Long-term Care Focus) data to link lagged (2018) indicators of nursing home payer mix, as indicated by the percentage of residents within each facility primarily insured by Medicaid.^25^ LTCFocus is sponsored by the National Institute on Aging through a cooperative agreement with the Brown University School of Public Health. We used the National Center for Health Statistics designations to classify whether facilities were located within a rural county.^26^

### Statistical Analysis

We first estimated the percentage of high-risk nursing homes (facilities ≤5 km from areas at or exceeding the 85th percentile of nationalized wildfire risk) using their geocoded addresses. We compared facility characteristics between nursing homes according to their wildfire exposure status. We also quantified the prevalence of critical emergency preparedness deficiencies for nursing homes, grouping states according to the CMS RO that they belong to: RO 6 (New Mexico), RO 8 (Mountain West), RO 9 (Pacific/Southwest), and RO 10 (Pacific Northwest). Because they supervise enforcement actions and compliance with federal emergency preparedness standards, we evaluated regional patterns of emergency preparedness and oversight corresponding to CMS RO designations.^4^

We evaluated cross-sectional associations between location within a wildfire-exposed area and the presence and count of emergency preparedness deficiencies using regionally stratified generalized estimating equations with binomial and negative binomial distributions, clustered by facility identification numbers, with robust SEs. To address potential confounding by facility or area characteristics, we adjusted for rurality, ownership, size, payer mix, and CMS ratings in the Health Inspection, Staffing, and Quality domains. Because of sparseness of the lowest scores in some subgroups, the CMS rating was dichotomized in adjusted analyses to indicate whether facilities received a high score (4-5 stars) in each domain. In sensitivity analyses, we reran the models for the 4 regional strata after increasing the severity of the wildfire exposure threshold to within 2.5 km from areas at or exceeding the 85th percentile of nationalized wildfire risk and within 2.5 km from areas at or exceeding the 95th percentile of nationalized wildfire risk.

Last, we conducted a survival analysis to estimate associations between wildfire exposure and the mean time to reinspection for the subset of facilities with a critical emergency preparedness deficiency. Because covariates in our adjusted model violated the proportional hazards assumption, we estimated the difference in time to first reinspection (in days) between exposed and unexposed facilities over a follow-up period of 15 months using restricted mean survival time analysis for each regional stratum. We calculated adjusted restricted mean survival time differences between exposed and unexposed nursing homes, adjusting for the number and average scope and severity of emergency preparedness deficiencies assigned during the first inspection within the observation period as well as the previously described area and facility characteristics.

We used a 2-tailed *P* value threshold of .05 to determine statistical significance. We conducted analyses using ArcGIS Pro, version 3.0 (Esri); Python, version 3 (Python Software Foundation); and Stata, version 17 (StataCorp). Data analysis was performed from October 10 to December 12, 2022.

## Results

The Figure displays the geocoded locations of the 2218 nursing homes in this study relative to areas with elevated risk of wildfire exposure. Of these nursing homes, 1219 (55.0%) were located within 5 km of areas at or exceeding the 85th percentile of nationalized wildfire risk. The Pacific/Southwest had the highest percentage of exposed nursing homes (870 of 1356 [64.2%]), followed by the Mountain West (215 of 408 [52.7%]) (eTable 2 in Supplement 1). As shown in Table 1, exposed facilities were smaller, with lower Medicaid share and proprietary ownership. The CMS ratings in the Health Inspection, Quality, and Staffing domains were comparable across the 2 groups. Similar proportions of exposed and unexposed facilities were located in rural counties across the overall sample. The Mountain West had the greatest percentage of rural facilities (97 of 408 [23.8%]), while the Pacific/Southwest had the lowest percentage of rural nursing homes (18 of 1356 [1.3%]). Less than 10% of nursing homes in New Mexico (5 of 64 [7.8%]) and the Pacific Northwest (23 of 390 [5.9%]) were located within rural areas. Exposed and unexposed facilities also underwent a similar number of LSC inspections during the observation period (mean [SD], 2.5 [0.6] vs 2.6 [0.6]).

**Figure.**
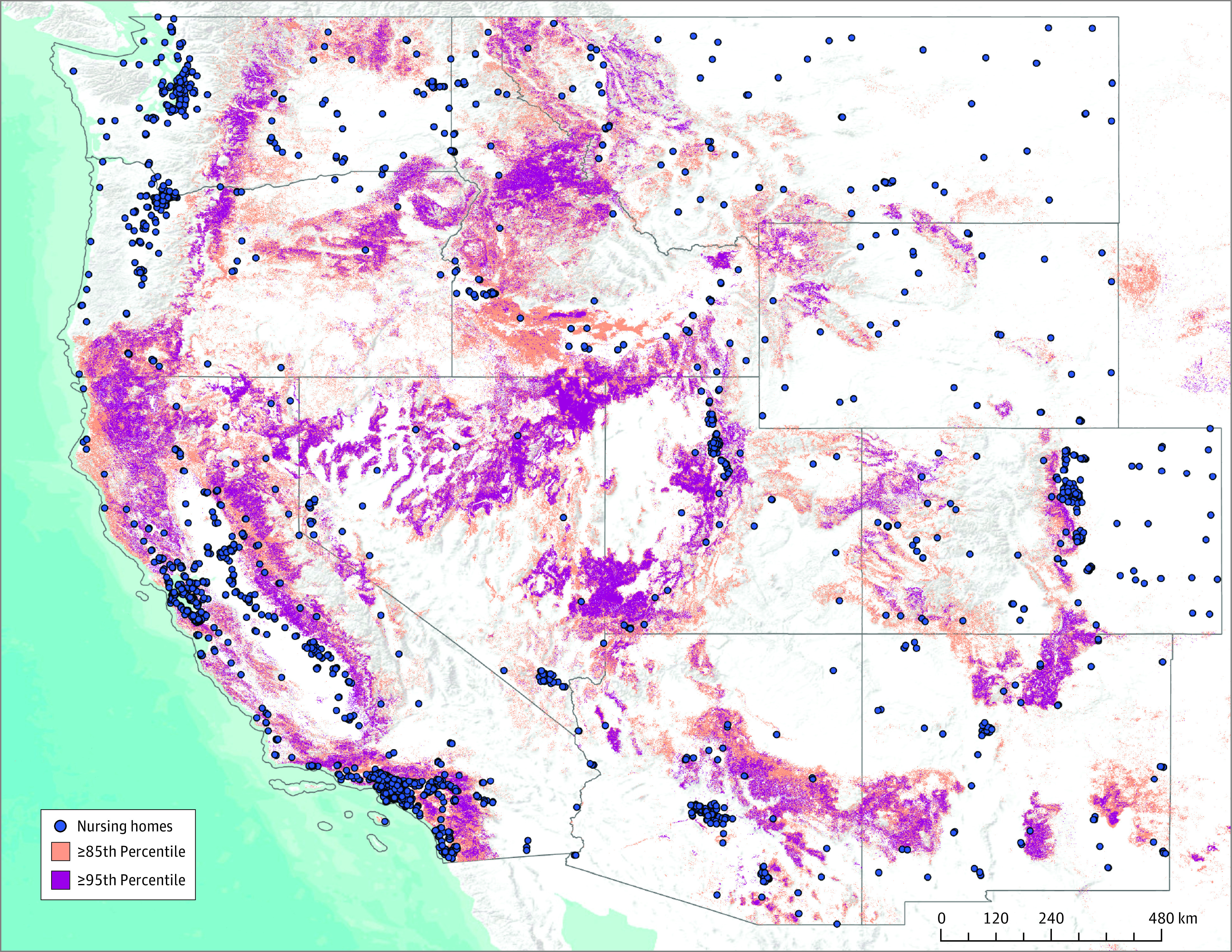
Nursing Home Exposure to Elevated Wildfire Risk in the Western US Geocoded locations of US Centers for Medicare & Medicaid Services–certified nursing homes are displayed as of December 1, 2017. Nursing home locations are superimposed on a visual adaptation of the US Wildfire Hazard Potential Index that summarizes the nationalized wildfire potential with 270-m resolution. Two thresholds of nationalized risk are displayed for areas at or exceeding the 85th and 95th percentiles.

**Table 1.** Sample Characteristics of Nursing Homes by Exposure to Wildfire Risk^a^

Characteristic	Overall sample (N = 2218)	Unexposed (n = 999)	Exposed (n = 1219)^b^	*P* value^c^
Proprietary ownership	1780 (80.3)	822 (82.3)	958 (78.6)	.03
CMS inspection rating^d^				
Health				
1	439 (19.8)	204 (20.4)	235 (19.3)	.57
2	518 (23.4)	234 (23.4)	284 (23.3)	
3	505 (22.8)	237 (23.7)	268 (22.0)	
4	501 (22.6)	218 (21.8)	283 (23.2)	
5	235 (10.6)	97 (9.7)	138 (11.3)	
Missing	20 (0.9)	9 (0.9)	11 (0.9)	
Quality				
1	114 (5.1)	51 (5.1)	63 (5.2)	>.99
2	242 (10.9)	111 (11.1)	131 (10.7)	
3	323 (14.6)	146 (14.6)	177 (14.5)	
4	430 (19.4)	194 (19.4)	236 (19.4)	
5	1084 (48.9)	484 (48.4)	600 (49.2)	
Missing	25 (1.1)	13 (1.3)	12 (1.0)	
Staffing				
1	58 (2.6)	21 (2.1)	37 (3.0)	.34
2	182 (8.2)	85 (8.5)	97 (8.0)	
3	623 (28.1)	291 (29.1)	332 (27.2)	
4	898 (40.5)	412 (41.2)	486 (39.9)	
5	380 (17.1)	159 (15.9)	221 (18.1)	
Missing	77 (3.5)	31 (3.1)	46 (3.8)	
No. of beds, mean (SD)	97.7 (50.8)	101.1 (52.3)	94.8 (49.3)	.004
Percentage of Medicaid share, mean (SD)	59.2 (26.1)	60.1 (24.4)	58.4 (27.4)	.013
Missing	64 (2.9)	31 (3.1)	33 (2.7)	
Rurality	143 (6.4)	64 (6.4)	79 (6.5)	.94
No. of inspections, mean (SD)	2.6 (0.6)	2.5 (0.6)	2.6 (0.6)	.07

Abbreviation: CMS, US Centers for Medicare & Medicaid Services.

^a^ Values are reported as the No. (%) of nursing homes unless indicated otherwise.

^b^ Exposed nursing homes are located within 5 km of an area at or exceeding the 85th percentile of nationalized wildfire risk.

^c^ Differences between exposed and unexposed nursing homes were tested using χ^2^ tests for categorical characteristics and unpaired *t* tests for continuous characteristics.

^d^ For the CMS 5-star rating system, higher scores indicate better performance.

eTable 3 in Supplement 1 provides the prevalence and number of critical emergency preparedness deficiencies by region and exposure status. The Pacific/Southwest had the highest percentage of both exposed (680 of 870 [78.2%]) and unexposed (359 of 486 [73.9%]) facilities with at least 1 critical emergency preparedness deficiency. The Mountain West had the largest difference in the percentage of exposed (87 of 215 [40.5%]) vs unexposed (47 of 193 [24.4%]) facilities with at least 1 critical emergency preparedness deficiency and the lowest prevalence of facilities with at least 1 deficiency across all regions. Facilities in the Pacific Northwest had the greatest mean (SD) number of deficiencies per nursing home (4.3 [5.4]).

Overall, the most common emergency preparedness deficiencies included failure to conduct emergency-related testing and exercise requirements, implement emergency and standby power systems, and address subsistence needs for staff and patients (eTable 1 in Supplement 1). As given in Table 2, the Pacific/Southwest had the highest percentage of facilities with failure to implement emergency and standby power systems (374 of 1356 [27.6%]), while the Pacific/Southwest and Pacific Northwest had the highest percentages of facilities that failed to address subsistence needs for staff and patients (230 of 1356 [17.0%] and 115 of 390 [29.5%]).

**Table 2.** Prevalence of Most Common Emergency Preparedness Deficiencies by CMS Regional Office

CMS LSC deficiency code	Description	Nursing homes with deficiency, No. (%)
New Mexico (n = 64)		
E-0007	Address patient/client population and determine types of services needed	27 (42.2)
E-0036	Establish emergency preparedness training and testing	24 (37.5)
E-0024	Establish policies and procedures for volunteers	17 (26.6)
E-0035	Provide family notifications of emergency plan	10 (15.6)
E-0015	Address subsistence needs for staff and patients	9 (14.1)
Mountain West (n = 408)		
E-0039	Conduct testing and exercise requirements	51 (12.5)
E-0004	Develop and maintain an emergency preparedness program	50 (12.3)
E-0026	Establish roles under a waiver declared by secretary	42 (10.3)
E-0024	Establish policies and procedures for volunteers	38 (9.3)
E-0037	Establish staff and initial training requirements	33 (8.1)
Pacific/Southwest (n = 1356)		
E-0039	Conduct testing and exercise requirements	405 (29.9)
E-0041	Implement emergency and standby power systems	374 (27.6)
E-0013	Develop emergency preparedness policies and procedures	298 (22.0)
E-0029	Develop a communication plan	260 (19.2)
E-0015	Address subsistence needs for staff and patients	230 (17.0)
Pacific Northwest (n = 390)		
E-0039	Conduct testing and exercise requirements	116 (29.7)
E-0015	Address subsistence needs for staff and patients	115 (29.5)
E-0037	Establish staff and initial training requirements	88 (22.6)
E-0026	Establish roles under a waiver declared by secretary	85 (21.8)
E-0018	Establish procedures for tracking staff and patients during an emergency	76 (19.5)

Abbreviations: CMS, US Centers for Medicare & Medicaid Services; LSC, Life Safety Code Inspection.

Table 3 provides the adjusted regional associations between wildfire exposure risk and the likelihood and number of critical emergency preparedness deficiencies. Exposed nursing homes had a greater likelihood of a critical emergency preparedness deficiency than unexposed facilities within the Mountain West (odds ratio, 2.12 [95% CI, 1.50-3.01]) and Pacific Northwest (odds ratio, 1.84 [95% CI, 1.55-2.18]). We observed a similar pattern with respect to the number of critical emergency preparedness deficiencies in the Pacific Northwest (rate ratio, 1.39 [95% CI, 1.06-1.83]). In contrast, associations were not observed for New Mexico or the Pacific/Southwest.

**Table 3.** Association Between Wildfire Exposure Risk and the Presence and Number of Emergency Preparedness Deficiencies^a^

Regional office	Presence of an emergency preparedness deficiency, adjusted odds ratio (95% CI)	No. of emergency preparedness deficiencies, adjusted rate ratio (95% CI)
New Mexico	1.07 (0.77-1.49)	1.25 (0.80-1.95)
Mountain West	2.12 (1.50-3.01)	1.54 (1.00-2.38)
Pacific/Southwest	1.00 (0.91-1.10)	1.01 (0.89-1.14)
Pacific Northwest	1.84 (1.55-2.18)	1.39 (1.06-1.83)

^a^ The reported associations are adjusted for rurality, ownership, size, payer mix, and US Centers for Medicare & Medicaid Services ratings in the Health Inspection, Staffing, and Quality domains.

Table 4 summarizes the regional mean differences in time between the first inspection and the subsequent LSC inspection for nursing homes with a critical emergency preparedness deficiency by exposure status. In the unadjusted analysis, exposed nursing homes had longer mean durations to reinspection than their unexposed counterparts. Exposed nursing homes in New Mexico experienced the longest absolute duration to reinspection (mean [SE], 406.1 [23.1] days) compared with other regions. Unexposed nursing homes in the Mountain West had the shortest absolute duration to reinspection (mean [SE], 275.9 [28.1] days) compared with other regions. The adjusted difference for the Mountain West was statistically significant, with exposed nursing homes reinspected a mean of 91.2 days (95% CI, 30.6-151.8 days) later than unexposed facilities.

**Table 4.** Differences in Time to Reinspection Between Exposed and Unexposed Nursing Homes With Emergency Preparedness Deficiencies^a^

Regional office	Time to reinspection, mean (SE), d		Difference, mean (95% CI), d	
	Exposed	Unexposed	Unadjusted	Adjusted
New Mexico	406.1 (23.1)	373.5 (15.3)	32.6 (−21.7 to 86.9)	23.0 (−12.2 to 58.3)
Mountain West	346.4 (14.8)	275.9 (28.1)	70.6 (8.3 to 132.9)	91.2 (30.6 to 151.8)
Pacific/Southwest	359.0 (2.4)	353.8 (3.1)	5.2 (−2.6 to 13.0)	7.0 (−0.8 to 14.8)
Pacific Northwest	381.3 (12.8)	376.0 (9.7)	5.4 (−26.2 to 36.9)	8.1 (−24.7 to 40.8)

^a^ Fifteen-month restricted mean survival time differences are presented. Adjusted differences account for the number of emergency preparedness deficiencies, the average scope and severity of emergency preparedness deficiencies, rurality, ownership, size, payer mix, and US Centers for Medicare & Medicaid Services 5-star ratings in the Health Inspection, Staffing, and Quality domains.

### Sensitivity Analysis

The magnitude and significance of associations were similar for the 2 alternative exposure definitions (eTable 4 in Supplement 1). Overlap between the point estimates for the first alternative definition and 95% CIs for the second did not support a dose-response association between greater exposure risk and emergency preparedness.

## Discussion

More than half of nursing homes in the continental western US are within 5 km of areas with elevated wildfire risk, heightening the importance of their emergency preparedness. Critical emergency preparedness deficiencies, including those focusing on competencies that are necessary to safely shelter residents in place or evacuate them when appropriate, were prevalent within each regulatory region.^27^ We observed geographic variation in the association between nursing home exposure and emergency preparedness. Contrary to expectations, we observed either no association or an increased likelihood and number of deficiencies for exposed facilities. Our findings suggest that high-risk facilities in the Mountain West and Pacific Northwest were more likely to have emergency preparedness deficiencies than their unexposed counterparts. Also contrary to our hypotheses, we observed either no association between exposure and the mean time to reinspection or paradoxically longer durations for exposed facilities. High-risk facilities in the Mountain West were reinspected, on average, 91.2 days later than lower-risk facilities.

The poorer emergency preparedness of high-risk nursing homes suggests that management and staff may be unaware of surrounding wildfire risk. The comparable, if not longer, time to reinspection for high-risk facilities with documented emergency preparedness deficiencies suggests that regulators may also be unfamiliar with local wildfire risk. The longer duration to reinspection for high-risk facilities in the Mountain West may be due, at least in part, to challenges in maintaining regulatory oversight in regions with a high concentration of rural areas. Although our models adjusted for rurality, data were not available on more specific factors that may correlate with rurality or the concentration of rural counties within a region, such as driving time between facilities and the prevalence of regulators. Greater than 20% of nursing homes in the Mountain West were located within rural areas, compared with less than 10% in other regions. In regions where nursing homes may be less accessible or regulatory staff may be less available, it would be prudent to prioritize high-risk nursing homes with outstanding deficiencies for reinspection.

Projected increases in community wildfire exposure^10^ heighten the importance of adequate emergency preparedness and increased inspection frequency for nursing homes with greater exposure risk. Considering the unique vulnerability of nursing home residents to external stressors, including environmental hazards, emergency preparedness should be better aligned with the likelihood of exposure to wildfire episodes. Prior research has shown significant increases in morbidity and mortality among residents of congregate care settings exposed to disasters.^28,29^ The CMS all-hazards approach to emergency preparedness guidance promotes comprehensive nursing home preparation for salient risks—ranging from infectious diseases to local environmental hazards.^30^ While this framework encourages nursing homes to engage with municipal disaster planning agencies, clear standards to enforce the strength or adequacy of these partnerships have not been developed.^3,30,31^ Understanding how partnerships with municipal disaster planning agencies could improve the sensitivity of both nursing home staff and regulators to pertinent hazards may better align emergency preparedness with local environmental risks. These partnerships may also aid in identifying prevalent regional exposures that could warrant tailored emergency preparedness standards. For example, updating building code guidance on acceptable ventilation standards could reduce residents’ exposure to particulate matter in wildfire-prone areas. Improved oversight of these partnerships by CMS ROs could expedite progress toward these ends.

### Limitations

This study has limitations. A standard distance-based evacuation threshold does not exist due to inherent challenges in predicting wildfire behavior and appropriate evacuation radii.^32,33^ Because municipal wildfire guidelines cite potential ember-induced secondary fires up to 16 km from the main fire front,^34^ our chosen distance thresholds (5 and 2.5 km) are conservative. In addition, our analysis focused on a single environmental exposure that is concentrated within the western US. As additional geospatial information becomes available, future research should incorporate multiple categories of environmental risk, such as flooding and earthquakes.

We cannot exclude the possibility that state surveyors were more vigilant in issuing emergency preparedness deficiencies based on perceived wildfire exposure risk. To address this limitation, we conducted sensitivity analyses that evaluated associations between emergency preparedness and increasingly severe exposure thresholds. We also evaluated the mean time to reinspection based on deficiency and exposure status for each nursing home. The results of our cross-sectional sensitivity analyses did not suggest a dose-response association between the severity of exposure risk and the likelihood of critical emergency preparedness deficiencies. Nor did the results of the survival analysis suggest that exposure status expedited time to reinspection for noncompliant facilities. Nonetheless, we could not fully account for differences in surveyors’ sensitivity to local environmental risk in our analyses.

Our evaluation assumes that administrative deficiencies are acceptable indicators of nursing homes’ emergency preparedness, but administrative preparedness may not always translate to effective emergency planning or response.^35^ We cannot exclude the possibility that some of the selected deficiencies are less germane to the execution of an effective emergency response plan. However, the select deficiencies included within the outcome definition are well aligned with national emergency preparedness guidance and prior literature linking preparedness to resident outcomes.^22,23,36^

The observational nature of this study did not permit us to evaluate potential mechanisms that could explain regional differences in the emergency preparedness of nursing homes at increased risk of wildfire exposure. Qualitative studies that evaluate regional variation in organizational or regulatory emergency preparedness practices could provide complementary information.

## Conclusions

Aligning nursing home emergency preparedness with local wildfire risk is important to resident safety. Regional variation in the emergency preparedness of nursing homes with an elevated risk of exposure to wildfire was observed in this study. Our findings suggest that nursing homes may benefit from enhanced enforcement of partnerships with local emergency planning agencies, which are better equipped to appraise environmental risks and call attention to nursing homes with disparate wildfire exposure risk. This would permit regulators to prioritize exposed nursing homes with outstanding, severe, or recurrent emergency preparedness deficiencies for reinspection and enhanced oversight. The CMS ROs are well positioned to accelerate progress toward nursing home emergency preparedness and regulatory oversight that are commensurate with surrounding environmental risks.

## Notes

Supplement 1. **eTable 1.** Frequency of Deficiency Codes for Western US Nursing Homes **eTable 2.** Prevalence of Exposed Facilities Under Different Exposure Thresholds **eTable 3.** Summary of Emergency Preparedness Deficiencies by US Centers for Medicare and Medicaid (CMS) Regional Office and Potential Wildfire Exposure **eTable 4.** Associations Between Wildfire Exposure and Emergency Preparedness Deficiencies for 2 Alternative Exposure Definitions

Supplement 2.
Data Sharing Statement
