# Supplementary material for: Assessment of Regional Nursing Home Preparedness for and Regulatory Responsiveness to Wildfire Risk in the Western US
Source: JAMA Netw Open. 2023 Jun 26;6(6):e2320207. doi: 10.1001/jamanetworkopen.2023.20207 (PMC10293909; doi:10.1001/jamanetworkopen.2023.20207)
Supplement: Supplement 1. — eTable 1. Frequency of Deficiency Codes for Western US Nursing Homes eTable 2. Prevalence of Exposed Facilities Under Different Exposure Thresholds eTable 3. Summary of Emergency Preparedness Deficiencies by US Centers for Medicare and Medicaid (CMS) Regional Office and Potential Wildfire Exposure eTable 4. Associations Between Wildfire Exposure and Emergency Preparedness Deficiencies for 2 Alternative Exposure Definitions [file jamanetwopen-e2320207-s001.pdf]

Supplementary Online Content

Festa N, Throgmorton KF, Davis-Plourde K, et al. Assessment of regional nursing home preparedness for and regulatory responsiveness to wildfire risk in the western US. *JAMA Netw Open*. 2023;6(6):e2320207. doi:10.1001/jamanetworkopen.2023.20207

eTable 1. Frequency of Deficiency Codes for Western US Nursing Homes

eTable 2. Prevalence of Exposed Facilities Under Different Exposure Thresholds

eTable 3. Summary of Emergency Preparedness Deficiencies by US Centers for Medicare and Medicaid (CMS) Regional Office and Potential Wildfire Exposure

eTable 4. Associations Between Wildfire Exposure and Emergency Preparedness Deficiencies for 2 Alternative Exposure Definitions

This supplementary material has been provided by the authors to give readers additional information about their work

© 2023 Festa N et al. *JAMA Network Open*.

eTable 1. Frequency of Deficiency Codes for Western US Nursing Homes

Deficiency Code	Description	Freq.	Percent of Nursing Homes with Deficiency
E-0039	Conduct [emergency-related] testing and exercise requirements.	580	26.1%
E-0041	Implement emergency and standby power systems.	436	19.7%
E-0015	Address subsistence needs for staff and patients.	379	17.1%
E-0026	Establish roles under a Waiver declared by secretary.	353	15.9%
E-0013	Develop Emergency Preparedness policies and procedures.	348	15.7%
E-0036	Establish emergency prep training and testing.	324	14.6%
E-0029	Develop a communication plan.	302	13.6%
E-0004	Develop and maintain an Emergency Preparedness Program (EP).	280	12.6%
E-0031	Provide emergency officials' contact information.	274	12.4%
E-0037	Establish staff and initial training requirements.	268	12.1%
E-0006	Conduct risk assessment and an All-Hazards approach.	266	12.0%
E-0035	Provide family notifications of emergency plan.	247	11.1%
E-0024	Establish policies and procedures for volunteers.	231	10.4%
E-0018	Establish procedures for tracking staff and patients during an emergency.	222	10.0%
E-0030	List the names and contact information of those in the facility.	219	9.9%
E-0009	Include a process for Emergency Preparedness collaboration.	212	9.6%
E-0007	Address patient/client population and determine types of services needed.	210	9.5%
E-0032	Provide primary/alternate means for communication.	184	8.3%
E-0023	Establish policies and procedures for medical documentation.	181	8.2%
E-0020	Establish policies and procedures including evacuation.	119	5.4%

© 2023 Festa N et al. *JAMA Network Open*.

Deficiency Code	Description	Freq.	Percent of Nursing Homes with Deficiency
E-0034	Provide a means of sharing information on occupancy/needs.	105	4.7%
E-0033	Establish methods for sharing information.	103	4.6%
E-0022	Establish policies and procedures for sheltering.	93	4.2%
E-0001	Establish an Emergency Preparedness Program (EP).	85	3.8%
E-0025	Create arrangements with other facilities to receive patients.	79	3.6%

© 2023 Festa N et al. *JAMA Network Open*.

eTable 2. Prevalence of Exposed Facilities Under Different Exposure Thresholds

**CMS Regional Office Grouping**	**States in Grouping**	**Total Facilities**	**≤5 Kilometers of High or Very High Risk** (n, %)	**≤2.5 Kilometers of High or Very High Risk** (n, %)	**≤2.5 Kilometers of Very High Risk** (n, %)
New Mexico	NM	64	22 (34.4)	17 (26.6)	11 (17.2)
Mountain West	CO, MT, UT, WY	408	215 (52.7)	123 (30.2)	47 (11.5)
Pacific/Southwest	AZ, CA, NV	1356	870 (64.2)	546 (40.3)	220 (16.2)
Pacific Northwest	ID, OR, WA	390	112 (28.7)	61 (15.6)	25 (6.4)

Abbreviations: CMS: Centers for Medicare & Medicaid Services

© 2023 Festa N et al. *JAMA Network Open*.

eTable 3. Summary of Emergency Preparedness Deficiencies by US Centers for Medicare and Medicaid (CMS) Regional Office and Potential Wildfire Exposure

Exposure by CMS Regional Office Grouping	No. Nursing Homes	No. (%) Nursing Homes with Critical EP Deficiency	Mean Number of Critical EP Deficiencies per Nursing Home (SD)	Critical EP Deficiency Range
**New Mexico**				
Unexposed	42	28 (66.7)	2.7 (2.9)	0-13
Exposed	22	17 (77.3)	3.0 (3.6)	0-12
**Mountain West**				
Unexposed	193	47 (24.4)	1.2 (3.2)	0-24
Exposed	215	87 (40.5)	1.5 (2.8)	0-15
**Pacific/Southwest**				
Unexposed	486	359 (73.9)	3.1 (3.8)	0-29
Exposed	870	680 (78.2)	3.3 (3.5)	0-26
**Pacific Northwest**				
Unexposed	278	171 (61.5)	3.2 (4.9)	0-31
Exposed	112	73 (65.2)	4.3 (5.4)	0-26

Abbreviations: CMS: Centers for Medicare & Medicaid Services; EP: Emergency Preparedness; SD: Standard Deviation

© 2023 Festa N et al. *JAMA Network Open*.

eTable 4. Associations Between Wildfire Exposure and Emergency Preparedness Deficiencies for 2 Alternative Exposure Definitions[a]

	Dichotomous (Primary) Outcome		Count (Secondary) Outcome	
	≤2.5 Kilometers of Areas with ≥85th Percentile Nationalized Wildfire Risk	≤2.5 Kilometers of Areas with ≥95th Percentile Nationalized Wildfire Risk	≤2.5 Kilometers of Areas with ≥85th Percentile Nationalized Wildfire Risk	≤2.5 Kilometers of Areas with ≥95th Percentile Nationalized Wildfire Risk
**CMS Regional Office Grouping**	Odds Ratio (95% CI)	Odds Ratio (95% CI)	Rate Ratio (95% CI)	Rate Ratio (95% CI)
New Mexico	1.14 (0.84, 1.57)	1.16 (0.84, 1.62)	1.18 (0.73, 1.88)	1.09 (0.65, 1.83)
Mountain West	1.36 (0.96, 1.93)	1.63 (1.05, 2.51)	1.26 (0.81, 1.97)	1.52 (0.88, 2.62)
Pacific/Southwest	0.97 (0.89, 1.07)	1.03 (0.91, 1.16)	0.95 (0.84, 1.06)	1.11 (0.95, 1.29)
Pacific Northwest	1.45 (1.12-1.87)	--	0.94 (0.69, 1.27)	0.85 (0.57, 1.27)

Abbreviations: CMS: Centers for Medicare & Medicaid Services; CI: Confidence Interval

a: The reported associations are adjusted for rurality, proprietary ownership, facility size, Medicaid share, CMS 5-Star Quality and Staffing Ratings.
-- indicates result omission due to poor model convergence (tolerance>1x10^-06)

© 2023 Festa N et al. *JAMA Network Open*.
