# Supplementary material for: Assessment of Regional Nursing Home Preparedness for and Regulatory Responsiveness to Wildfire Risk in the Western US
Source: JAMA Netw Open. 2023 Jun 26;6(6):e2320207. doi: 10.1001/jamanetworkopen.2023.20207 (PMC10293909; doi:10.1001/jamanetworkopen.2023.20207)
Supplement: Supplement 2. — Data Sharing Statement [file jamanetwopen-e2320207-s002.pdf]

Data Sharing Statement

Festa. Assessment of Regional Nursing Home Preparedness for and Regulatory Responsiveness to Wildfire Risk in the Western US. *JAMA Netw Open*. Published June 26, 2023. doi:10.1001/jamanetworkopen.2023.20207

Data
Data available: No

Additional Information
Explanation for why data not available: All data used for this study is accessible to the public and cited for ease of access.
